# Resettlement in New Zealand

**Published:** 1920-11-27

**Authors:** 


### Resettlement in New Zealand.

We are indebted to a correspondent, "A. R. P.," for an interesting account of the difficulties encountered by New Zealand War Sisters in resettling after their return home. Apart from those retained for service in the various military hospitals, it appears from the annual report of the Director-General of Hospitals, that a good many have not resumed nursing work. Special courses have been arranged by the Repatriation Board, and of these some nursing sisters have taken advantage. Massage has attracted a good many, and twelve-month courses at the Otago University and the Dunedin Hospital have proved popular. Hospital Boards have not in all cases behaved well or even kept to the undertakings they gave when the call for sisters in the war was first heard. The Director-General rightly declares that these sisters, being returned soldiers, were entitled to special consideration in making appointments on their staffs, but Boards have not realised that the great experience these women have gained would be of value to the hospitals. The notorious instance of the Board which refused to allow its matron to complete her service in the Army and superseded her with six months' leave on pay is severely commented on. "Seven of the military sisters underwent very thorough training in the administration of anæsthetics in the newest methods, and had very extensive practice in casualty clearing-stations and stationary hospitals. So far one only of these sisters has been given the opportunity of carrying on the work."

